# A malaria death due to an imported *Plasmodium falciparum* infection in Sri Lanka during the prevention of re-establishment phase of malaria

**DOI:** 10.1186/s12936-023-04681-5

**Published:** 2023-08-24

**Authors:** Shilanthi Seneviratne, Deepika Fernando, Pubudu Chulasiri, Kumudu Gunasekera, Nethmini Thenuwara, Champa Aluthweera, Anula Wijesundara, Rohini Fernandopulle, Kamini Mendis, Rajitha Wickremasinghe

**Affiliations:** 1 Anti Malaria Campaign, Ministry of Health, Colombo, Sri Lanka; 2 Department of Parasitology, Faculty of Medicine, University of Colombo, Colombo, Sri Lanka; 3 142/2A, Vijaya Kumaratunge Road, Colombo 5, Colombo, Sri Lanka; 4 Faculty of Medicine, General Sir John Kotelawala Defense University, Ratmalana, Sri Lanka; 5 Faculty of Medicine, University of Kelaniya, Kelaniya, Sri Lanka

**Keywords:** Imported malaria, Death, Delayed diagnosis, Case fatality, Prevention of re-establishment, Travel health

## Abstract

**Background:**

Sri Lanka has maintained a rigorous programme to prevent the re-establishment of malaria ever since the disease was eliminated in October 2012. It includes efforts to sustain case surveillance to ensure early diagnosis and management of malaria. Yet, in April of 2023 the death occurred of an individual with imported malaria.

**Case presentation:**

The deceased was a 37-year-old Sri Lankan male who returned to Sri Lanka on the 10th of April after a business trip to several countries including Tanzania. He was febrile on arrival and consulted three Allopathic Medical Practitioners in succession in his home town in the Western Province of Sri Lanka, over a period of 5 days starting from the very day that he arrived in the country. Malaria was not tested for at any of these consultations and his clinical condition deteriorated. On the evening of 14th of April he was admitted to the medical intensive care unit of a major private hospital in the capital city of Colombo with multiple organ failure. There, on a request by the treating physician blood was tested for malaria and reported early the next morning as *Plasmodium falciparum* malaria with a high parasitaemia (> 10%). The patient died shortly after on the 15th of April before any anti-malarial medication was administered. The deceased had been a frequent business traveller to Africa, but with no past history of malaria. He had not taken chemoprophylaxis for malaria on this or previous travels to Africa.

**Discussion:**

The patient’s *P. falciparum* infection progressed rapidly over 5 days of arriving in Sri Lanka leading to severe malaria without being diagnosed, despite him seeking healthcare from three different Medical Practitioners. Finally, a diagnosis of malaria was made on admission to an intensive care unit; the patient died before anti-malarial medicines were administered.

**Conclusions:**

This first death due to severe *P. falciparum* malaria reported in Sri Lanka after elimination of the disease was due to the delay in diagnosing malaria.

## Background

Sri Lanka is a malaria-free country certified by the World Health Organization (WHO) in 2016 and is now in the phase of Prevention of Re-establishment (PoR) of malaria. Since the beginning of the PoR phase in 2013, till the end of 2022, 470 malaria cases were reported in the country all of them being imported infections with the exception of an introduced case of malaria reported in 2018 and a transfusion-induced case reported in 2021 [1, 2]. Two hundred and twelve of these cases were acquired in Africa, 152 in India and the rest 84 in other countries.

Between 1955 and 2022, 103 countries and territories have succeeded in eliminating malaria globally [3]. However, imported malaria cases continue to be reported due to increased international travel and population movement, which closely links these countries with malaria endemic areas of the world [4, 5]. A meta-analysis on the geography of imported malaria in non-endemic countries over a 10-year period (2005–2015), showed that the highest number of imported malaria cases were reported from France, UK and USA [6]. A more recent study carried out in 2018, has confirmed 8,347 imported malaria cases in Europe with over 50% of them being reported from France, UK, Germany, and Spain [7]. Currently, migrants from endemic countries constitute a high proportion of imported malaria cases in non-endemic countries [8].

Imported malaria cases pose challenges for diagnosis and management as malaria is not a frequently encountered disease for many physicians in non-endemic areas [9, 10]. Delays in diagnosis of malaria, particularly in non-immune travelers are associated with a higher mortality risk [11, 12]. Further, with *Anopheles* vectors still present in many non-endemic countries, delayed treatment of imported cases can also increase the risk of secondary transmission [2, 13]. Sri Lanka has maintained a rigorous programme to prevent the re-establishment of malaria since the disease was eliminated in October 2012. Case surveillance plays a key role enabling early diagnosis and management. In Sri Lanka, over 90% of malaria cases are diagnosed by passive case detection [14] and the rest by active case detection, both proactive and reactive case detection. Sustaining awareness of medical practitioners on malaria, now a rare disease, is also a key function of the Anti Malaria Campaign (AMC). When a case of (imported) malaria is detected, the AMC investigates the case, conducts both reactive parasitological and entomological surveillance in the area soon after the case is reported, and takes action on the basis of surveillance data.

Despite a rigorous programme to prevent the re-establishment of malaria and malaria deaths, the death of an individual with imported malaria in April 2023 is described in this paper, the first malaria death to be reported after the disease was eliminated. Historically, when malaria was endemic in Sri Lanka, malaria deaths were not uncommon, and nearly all were due to *P. falciparum,* which, at most times accounted for less than half of all malaria cases, the dominant species having been *Plasmodium vivax.* As the malaria incidence declined in Sri Lanka from 1999 onwards, the number of deaths due to indigenous malaria also declined simultaneously from 102 in 1999 to 76 in year 2000. Thereafter, a 68% reduction in malaria case incidence was recorded between years 2000 and 2001 and the incidence continued to decline further in subsequent years [15]. Correspondingly, malaria deaths also continued to decline with a total of 88 indigenous malaria deaths reported between 2001 and 2004. The last death due to malaria was reported in 2007 in a patient who had acquired malaria overseas. The malaria death reported in this paper is the first since elimination and malaria-free certification. This paper focuses on aspects relating to the prevention of malaria in a traveller, and timely diagnosis of malaria to prevent severe malaria and death, and does not delve deeply into the clinical management aspects of the case.

## Case presentation

The deceased was a 37-year-old Sri Lankan male, a gem businessman by profession and a resident of Beruwala, a coastal town located 55 km South of Sri Lanka’s capital Colombo along the south-western coastline. He left Sri Lanka on the 22nd of February 2023 on a business trip. He visited Thailand for 30 days, United Arab Emirates (UAE) for 5 days and finally Tanzania where he spent the last 15 days of his trip and returned to Sri Lanka on the 10th of April 2023.

On the day of his arrival in Sri Lanka on the 10th April he was febrile and the same evening he visited a General Practitioner in his home town, Beruwela. He was reportedly prescribed antibiotics, to which he did not respond and experienced a worsening of symptoms the next day with vomiting and joint pains. Due to the severity of the illness and development of abdominal pain, he visited another General Practitioner in the Beruwela area on the 12th of April where he was once again prescribed antibiotics. The patient had not volunteered the history of his travel at the consultations. Malaria was not tested for by either general practitioners.

**Fig. 1:**
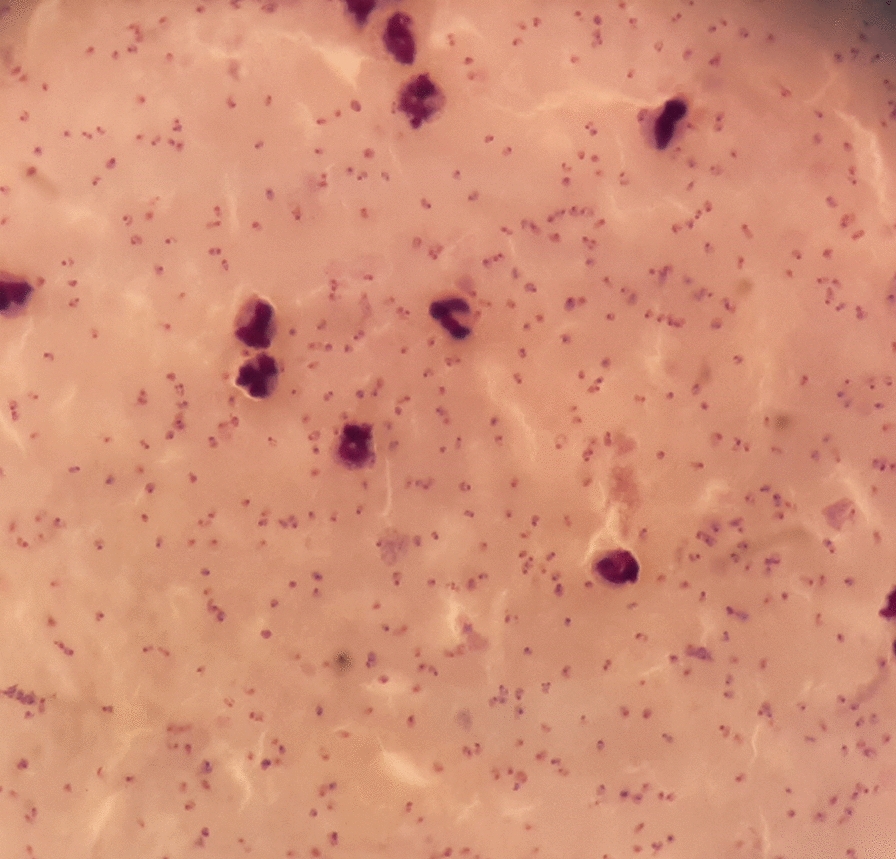
Giemsa stained thick blood smear (× 1000). Multiple *Plasmodium falciparum* rings

**Fig. 2:**
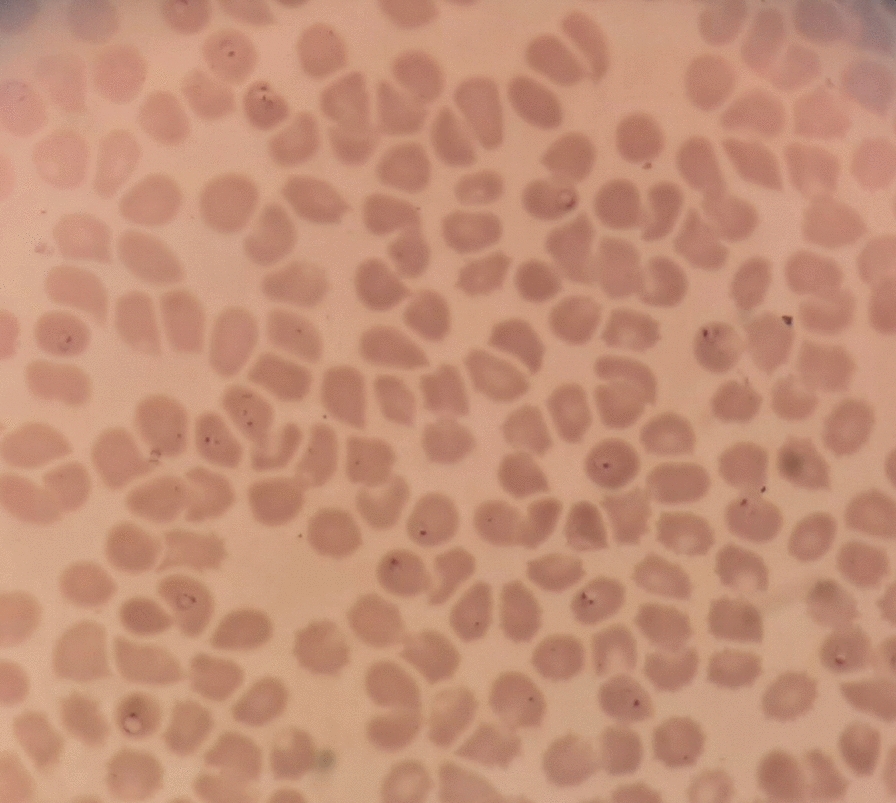
Giemsa stained thin blood smear (× 1000). Multiple *Plasmodium falciparum* rings

By the 13th of April, due to a deterioration of his condition, he contacted a third medical practitioner and the next morning, 14th April, he was jaundiced, complained of epigastric and right hypochondrial pain and was short of breath. All his consultations have been with registered allopathic medical practitioners. On that day, an ultrasound scan was done on him at a medical centre in Beruwela, which revealed splenomegaly and gall bladder oedema. On the advice of the Radiologist he was admitted to a private hospital in Colombo by 5.30 pm the same day (14th April) but due to high fever, dyspnea, an oxygen saturation of 86% the attending physician transferred him to a larger private hospital in Colombo at 7.00 pm, where he was admitted to the medical intensive care unit (ICU) there. On examination he was in acute renal failure and acute respiratory distress confirmed by chest X-ray. A request was made by the physician for examination of blood for malaria parasites on admission. The blood report issued by the hospital based on microscopy early the next morning (15thApril) was positive for *P. falciparum* malaria with a high parasitaemia (> 10%) (Figs. 1 and 2), and a severe thrombocytopenia (platelet count 30,000/mm^3^) with evidence of haemoconcentration. A rapid diagnostic test (RDT) was not performed, although RDTs were available in the ICU. The patient died on the same day within a hour of the malaria microscopy report being available and before any anti-malarial medication was administered. The cause of death was recorded as Acute Respiratory Distress Syndrome with high falciparum parasitaemia (> 10%).

The diagnosis of *P. falciparum* malaria was later confirmed by the central laboratory of the AMC on a blood smear which was taken at the time of admission. Parasite ring stages were present at a high density (839,638 parasites/μL). Rapid Diagnostic Tests based on both HRP-2 antigen and pLDH performed on this blood sample retrospectively also tested positive for *P. falciparum*.

Based on discussions with the patient’s relatives and friends following his death, it was revealed that this individual was a frequent business traveller to Africa, with a history of having visited Tanzania 3–4 times every year from 2013 onwards, excluding the periods of travel restrictions due to Covid-19. Reportedly he has not had any previous malaria infections. He has not used chemoprophylaxis for malaria on this or previous travels to Africa, but he used mosquito bite preventive measures, such as the application of repellents and sleeping under mosquito nets. The relatives of the deceased also mentioned that he was aware of malaria chemoprophylactic treatment being given free-of-charge by the AMC, but that he did not take them because he was concerned about the adverse effects of these medications. The community of gem traders in Beruwala to which the deceased belonged has been the source of several imported malaria cases in the past. The first two cases of imported malaria from this community since malaria was eliminated, was reported in 2015, one of them being a case of severe malaria which was successfully treated. Thereafter five imported malaria cases were reported from this community in Beruwala in each of the years 2016 and 2017. Following this, eight malaria cases were reported from the community between 2018 and 2022.

## Discussion

The report here is of the first malaria death to have occurred in the country since malaria was eliminated in 2012. The deceased individual was a member of a community of businessmen engaged in the gem trade in Beruwala, who travel frequently to African countries for the trade. He is likely to have contracted the *P. falciparum* infection in Tanzania, where the last 15 days of his journey was spent before arriving in Sri Lanka. Tanzania is amongst the ten countries with the highest number of malaria cases and deaths, accounting for 3% of the global cases and deaths with *P. falciparum* responsible for 96% of all malarial infections [16]. The other two countries he visited are either very lowly endemic (Thailand) [17] or free of malaria (United Arab Emirates) [3]. It is not known exactly when his malaria episode began as he was febrile on arrival in Sri Lanka; his malaria infection may have been clinically patent even whilst he was in Tanzania, but being a non-immune adult, it is entirely possible that he first became clinically ill on the day he arrived in Sri Lanka and that the infection progressed rapidly to reach a high parasite count within a span of a few days. He died of severe malaria with a very high blood parasite count and multiple organ dysfunction within 5 days of arrival in the country.

Malaria was not tested for as a possible cause of fever at any of the three medical consultations he made in the five days that transpired between his arrival in the country and death, until his last consultation on the eve of his death. By that time his clinical condition had deteriorated to a state of advanced multiple organ failure and the patient died within less than an hour of making a malaria diagnosis. Delayed diagnosis of malaria, particularly in travelers is associated with a high mortality rate [11, 12], possibly because they tend to be non-immune adults.

When malaria ceases to be endemic and becomes a rare disease in a country as it is now in Sri Lanka, physicians fail to include it in the differential diagnosis of fevers, for which many other highly prevalent febrile diseases such as dengue, other viral infections take precedence. Consequently, delays in diagnosing malaria and the associated high case fatality rates are an acknowledged risk in malaria non-endemic countries. The need to maintain the vigilance of health care providers on malaria in countries where malaria is not endemic is a major challenge and has been repeatedly stressed [10, 14, 18, 19]. In Iran, which is very near to eliminating malaria, an online tool was developed to evaluate the practice of healthcare providers in relation to the correct management of suspected malaria patients [20]. Reported case fatality rates of imported malaria in high-income malaria non-endemic countries have been as high as 1% [21–23]. In the UK, between 1987 and 2006, 39,302 cases of confirmed malaria were reported of which 25,054 cases were due to *P.falciparum* malaria. There were 191 malaria-associated deaths, of which 184 were associated with *P. falciparum*, giving a case fatality of 0.73%. The other seven deaths were associated with non-falciparum malaria (four from *P. vivax* and one each from *Plasmodium ovale* and *Plasmodium malariae*) [24, 25]. Geo-sentinel, a global clinician based sentinel surveillance system, reported 5689 individuals to have acquired imported malaria between January 2003 to June 2016 with 76% of them been diagnosed with a single *P. falciparum* infection. Four hundred and forty four of these travellers (92% who acquired the infection from sub-Saharan Africa) developed severe malaria of whom 12 died [26]. USA traveller surveillance findings from 2016 reports that 85% of severe cases imported to the USA were *P. falciparum* and all the severe malaria infections were acquired in sub-Saharan Africa. Most malaria infections were diagnosed amongst migrants who were permanently settled in non-endemic countries, but originating from malaria endemic areas and visiting friends and relatives [27]. Since Sri Lanka eliminated malaria in 2012 it has reported 470 imported malaria cases, 211 of them due to *P. falciparum*. This being the first and only death due to malaria reported so far in the country since malaria was eliminated, the case fatality rate of imported malaria in Sri Lanka thus far amounts to 0.21%, and 0.47% for *P. falciparum* malaria alone. These rates are somewhat lower than the reported average from most malaria non-endemic countries, possibly because being a tropical country at a very high risk of malaria being re-established through imported infections, rigorous measures are being taken to both prevent malaria in travelers as well as to reduce the delays in diagnosing imported cases.

The AMC being aware of the risks of delayed malaria diagnosis, monitors the time to a malaria diagnosis in every case of malaria since malaria was eliminated—both the time from the onset of the illness to the first contact with the health system, and the time from the first contact with the health system to a confirmed malaria diagnosis [14, 28]. Measures are being taken to keep the medical community aware of the need to elicit travel histories from febrile patients and request a malaria test through regular communications via Small Message Service (SMS), lectures and communications through the medical associations, and professional colleges, although not always with the desired results, as in this particular case. This is in accordance with current thinking that alertness of the general health services to suspected malaria (vigilance) needs to be maintained everywhere, while health education is rational only if targeting high-risk sub-populations [19]. Malaria diagnostic facilities by way of quality microscopy and RDTs are also being made widely available in Sri Lanka in both the public health sector with approximately 350 trained public health laboratory technicians in strategic locations across the country, and in the private health sector. A 24- hour hotline is available for reporting suspected malaria cases, through which guidance and assistance is provided on diagnosis and treatment of malaria. Therefore, it is not likely that non-testing for malaria by three medical practitioners in this case was due to the inability or difficulties to access malaria diagnostic facilities.

This particular community to which the deceased belonged to has long been the focus of AMCs health education campaigns, with repeated highly focused educational and awareness raising campaigns on malaria preventive behaviour, with an emphasis on the need to obtain chemoprophylactic medicines prior to travelling to malaria endemic countries, sometimes in collaboration with community and religious leaders. Yet, another key strategy which AMC implements during the POR phase of malaria to ensure early diagnosis of imported malaria is periodic screening of population groups that are at high risk of imported malaria such as the community to which the deceased person belonged. The last awareness raising programme in this community was carried out in August 2022 for a group of 200 attendees, and 16 eligible persons were screened for malaria and all reported negative. The previous programme was conducted in 2021 for the leaders of the Gem Traders Association, after which two important decisions were made and acted upon. One was to maintain stocks of chemoprophylactic medicines for travelers in the local Government Hospital in Beruwala, and the other was to provide long-lasting insecticidal nets to frequent travellers in this community, both free of charge. In 2022 and the first 4 months of 2023, 150 and 27 travellers, respectively, from this very community of gem traders in Beruwala alone received chemoprophylactic anti-malarials from AMC headquarters or the Regional Malaria Officer (RMO). However, this particular patient had not obtained malaria chemoprophylaxis. Thus, this unfortunate malaria death may point to the deceased being a non-compliant person in a community, which is otherwise being kept informed of the malaria risk during overseas travel as a high surveillance focus of the AMC.

On the same day the patient passed away, the Anti Malaria Campaign convened an emergency meeting including members of the Technical Support Group [29] to review information relating to this death and to discuss a formal investigation of this death. Immediately after, an investigation into the death was initiated by the Ministry of Health Sri Lanka.

Reactive parasitological surveillance was commenced immediately after diagnosis of malaria in this patient. One travel contact who arrived with the deceased from the same destination (Tanzania) was screened and found to be negative for malaria. The standard operating procedure of the AMC is to screen individuals in the household and neighbourhood within a one kilometre radius of the index case, if the index case had been in the country for at least 7 days (primary parasitological surveillance) for the purpose of detecting any malaria infections from which the patient could have acquired the disease locally, the minimum incubation period of *P. falciparum* being 7 days. Primary surveillance was not indicated in this case because the patient presented with fever on arrival in the country and was diagnosed with malaria within 5 days of disembarkation. Reactive entomological surveillance to assess the possibility of onward transmission of malaria from this patient commenced within 48 h of diagnosis of the index case within a one kilometre radius around the residence of the deceased. Indoor hand collections, human landing night collections and larval surveys were negative for the primary vector, *Anopheles culicifacies* and secondary vectors. Cattle-baited trap collections were negative for the primary vector, but there were low densities of *Anopheles vagus* and *Anopheles tessellatus* considered to be secondary vectors in the country.

In the absence of malaria vectors and as the residence of the deceased was located in a malaria non-endemic area, the probability of onward transmission of disease was minimal and therefore secondary parasitological screening was also not carried out on accordance with standard operating procedures of the AMC. However, a number of proactive surveillance operations were carried out by the AMC to screen high-risk populations in this area within the next two to three weeks (between 16^th^ April and 3^rd^ of May 2023). 242 individuals who had returned from malaria endemic countries were identified and screened for malaria. All travellers tested negative.

Along with the surveillance activities, awareness programmes were carried out for curative staff of the hospitals and private practitioners in the area that this community of gem traders sought treatment from. Awareness programmes were also carried out for the preventive public health staff at all Medical Officer of Health Offices. Communications were initiated with the Grama Niladhari (the village leaders) requesting referral of persons who travel to African countries to obtain chemoprophylaxis prior to departure in the future. Media briefings and televised discussions were held to increase general public awareness on malaria.

This unfortunate event draws attention to an alarming trend of malaria fast becoming a forgotten disease, with medical practitioners failing to diagnose malaria in time to save lives. There is also a need for the AMC to broaden the coverage and increase the intensity of its focused advocacy campaigns to keep the country free of malaria and to prevent malaria deaths.

## Conclusions

The reported malaria death of an adult traveler from Sri Lanka on his return from a malaria endemic country may have been the result of failure to suspect, diagnose and treat malaria by the 3 medical practitioners he consulted in the 5 days preceding his death. The patient himself not having divulged the history of overseas travel may have contributed to the delay in diagnosis. The risk of his contracting the malaria infection may also have been reduced had he complied with the advice being disseminated by the Anti Malaria Campaign to his community, and taken chemoprophylaxis. In a highly connected world where travel is rampant, this report calls for international travel health to be placed high on the agenda of all countries free of malaria for medical training and practice if malaria deaths in travelers are to be avoided. It is also a reminder of the high risk of malaria re-establishing in countries that have successfully eliminated malaria, particularly those in the tropical zone and therefore highly receptive to malaria. In such countries as Sri Lanka, a rigorous programme to prevent the re-establishment of malaria needs to be sustained to keep the disease at bay.

## Data Availability

The datasets generated and/or analysed in this publication are not publicly available due to the fact that they belong to the Ministry of Health, Sri Lanka. Clarifications regarding data can be made through Dr. Champa Aluthweera, Director of the Anti Malaria Campaign, Sri Lanka who is an author of this publication.

## Abbreviations

AMC: Anti Malaria Campaign
HRP-2: Histidine Rich Protein 2
pLDH: Plasma Lactate Dehydrogenase
PoR: Prevention of re-establishment
RMO: Regional Malaria Officer
RDT: Rapid Diagnostic Tests
SMS: Short Message Service
WHO: World Health Organization
